# Pathophysiology and mechanism of long COVID: a comprehensive review

**DOI:** 10.1080/07853890.2022.2076901

**Published:** 2022-05-20

**Authors:** D. Castanares-Zapatero, P. Chalon, L. Kohn, M. Dauvrin, J. Detollenaere, C. Maertens de Noordhout, C. Primus-de Jong, I. Cleemput, K. Van den Heede

**Affiliations:** Centre Administratif du Botanique, Belgian Health Care Knowledge Centre (KCE), Brussels, Belgium

**Keywords:** Long COVID, post-COVID-19 condition, COVID-19, pathology, physiology

## Abstract

**Background:**

After almost 2 years of fighting against SARS-CoV-2 pandemic, the number of patients enduring persistent symptoms long after acute infection is a matter of concern. This set of symptoms was referred to as “long COVID”, and it was defined more recently as “Post COVID-19 condition” by the World health Organization (WHO). Although studies have revealed that long COVID can manifest whatever the severity of inaugural illness, the underlying pathophysiology is still enigmatic.

**Aim:**

To conduct a comprehensive review to address the putative pathophysiology underlying the persisting symptoms of long COVID.

**Method:**

We searched 11 bibliographic databases (Cochrane Library, JBI EBP Database, Medline, Embase, PsycInfo, CINHAL, Ovid Nursing Database, Journals@Ovid, SciLit, EuropePMC, and CoronaCentral). We selected studies that put forward hypotheses on the pathophysiology, as well as those that encompassed long COVID patients in their research investigation.

**Results:**

A total of 98 articles were included in the systematic review, 54 of which exclusively addressed hypotheses on pathophysiology, while 44 involved COVID patients. Studies that included patients displayed heterogeneity with respect to the severity of initial illness, timing of analysis, or presence of a control group. Although long COVID likely results from long-term organ damage due to acute-phase infection, specific mechanisms following the initial illness could contribute to the later symptoms possibly affecting many organs. As such, autonomic nervous system damage could account for many symptoms without clear evidence of organ damage. Immune dysregulation, auto-immunity, endothelial dysfunction, occult viral persistence, as well as coagulation activation are the main underlying pathophysiological mechanisms so far.

**Conclusion:**

Evidence on why persistent symptoms occur is still limited, and available studies are heterogeneous. Apart from long-term organ damage, many hints suggest that specific mechanisms following acute illness could be involved in long COVID symptoms.

**KEY MESSAGES**
- Long-COVID is a multisystem disease that develops regardless of the initial disease severity. Its clinical spectrum comprises a wide range of symptoms.
- The mechanisms underlying its pathophysiology are still unclear. Although organ damage from the acute infection phase likely accounts for symptoms, specific long-lasting inflammatory mechanisms have been proposed, as well.
- Existing studies involving Long-COVID patients are highly heterogeneous, as they include patients with various COVID-19 severity levels and different time frame analysis, as well.

## Introduction

At the early stages of the COVID-19 pandemic, whose outbreak was officially declared in March 2020, it was recognized that the effects of SARS-CoV-2 (Severe Acute Respiratory Syndrome Coronavirus 2) likely vary from an asymptomatic infection to a multi-system disease. While most patients recovered in the weeks following acute infection, evidence quickly emerged that some people reported persistence or appearance of a wide variety of symptoms with variable intensity, regardless of the initial disease severity [1,2]. The term “long COVID” was introduced in May 2020 [3].

Patients with persisting symptoms constitute a very heterogeneous group. Initially, there was no globally accepted definition of long COVID. In December 2020, the National Institute for Health and Care Excellence (NICE) proposed a definition that was based on the time elapsed from the acute disease, when symptoms unexplained by an alternative diagnosis were still being reported [4]. The distinction was then made between “ongoing symptomatic COVID-19”, which applied to patients reporting symptoms at between 4 to 12 weeks following acute COVID-19, whereas “post-COVID-19 syndrome” was applied to those still experiencing symptoms 12 week after illness onset. In October 2021, the World Health Organization (WHO) proposed a consensus definition for what they referred to as “Post COVID-19 condition”. The condition was defined as the presence of symptoms lasting for at least 2 months in individuals with a history of probable or confirmed SARS-CoV-2 infection [5]. According to the WHO, this condition usually manifests 3 months from the onset of acute illness, yet cannot be explained by an alternative diagnosis. It is now clear that long COVID has become a meaningful public health concern, given that it now affects millions of people worldwide. The Office for National Statistics in the UK estimated that the prevalence of symptoms remaining following 12 weeks ranged from 3% to 11.7%, with a substantial deleterious impact on social and professional life, and day-to-day activities, as well [4].

Because the reported complaints can overlap with several other conditions that are not specifically related to COVID-19 (post-intensive care syndrome or exacerbation of pre-existing health conditions), substantial uncertainty remains as to why long-lasting symptoms occur. Intrinsically, people can present symptoms following organ damage that has developed during the acute illness phase, while others experience new symptoms in the aftermath of a mild infection, without any evidence of acquired organ or tissue damage [6–8].

Owing to both its emerging nature and complexity, a better knowledge of long COVID-related underlying mechanisms is paramount, as it could be instrumental in guiding further research. This systematic review explores the possible pathophysiological mechanisms underlying lingering symptoms after COVID-19. We made a distinction between mechanisms leading to organ dysfunction-related symptoms, and the pathophysiology that was presumably not associated with organ dysfunction upon acute illness.

## Methods

### Identification of studies

A scoping search was conducted in PubMed, which helped identify relevant articles. A search query was then developed with the assistance of a medical information specialist. Considering the topic specificities (recent topic, no clear concept, and several synonyms), a pure keyword-based strategy was to be conducted in the bibliographical databases. In addition, full text databases and pre-print registries were included in the sources to search, which a strategy adapted to each source.

In February 2021, a systematic search was conducted involving 11 sources (Cochrane Library, JBI EBP Database, Medline, Embase, PsycInfo, CINHAL, Ovid Nursing Database, Journals@Ovid, SciLit, EuropePMC, and CoronaCentral), which was then updated in late May 2021. Additional references were retrieved from diverse sources, including external experts, citation searches, and update searches in Pubmed, based on a simplified strategy (until 09 August 2021). Full methodological details were provided in the Belgian Health Care Knowledge Centre (KCE) report 344 [9].

### Study selection and data extraction

The selection of studies was independently conducted by an information specialist (PC) and a researcher (DCZ). The inclusion criteria were formulated by using the PEOD scheme (Population/Exposure/Outcome/Design), as follows: (1) studies putting forward hypotheses on the pathophysiological mechanisms (O) that could contribute to persisting symptoms (P) after COVID-19 (E); (2) studies that included long COVID patients in their research process. The study designs (D) were case series, systematic reviews, cohort studies, and experimental studies without any restriction concerning the included patient number. Languages were restricted to English, French, Dutch, and Spanish. Exclusion criteria were as follows: (1) studies that exclusively focussed on acute mechanisms; (2) studies that put forward hypotheses on long-term putative consequences like neurodegenerative diseases or cancers; (3) case reports. Articles were excluded if the content was essentially focussed on acute pathophysiological mechanisms underlying the acute infection phase. However, articles that discussed the contribution of early pathophysiological disturbances resulting in chronic symptoms were included, too.

### Quality assessment

Given that the majority of articles described hypotheses or exploratory investigations based on a translational approach with limited sample sizes, a classical critical appraisal, such as performed for clinical trials, could not be not undertaken for this review.

### Data analysis

A distinction was made between studies describing one or more hypotheses on the underlying pathophysiology of long COVID, and those analysing patient data. Both article types were analysed separately and classified by organ system and symptoms. The immune system was considered an all-encompassing system, given that it can involve many types of symptoms pertaining to various organs.

## Results

### Search results

**Figure 1.**
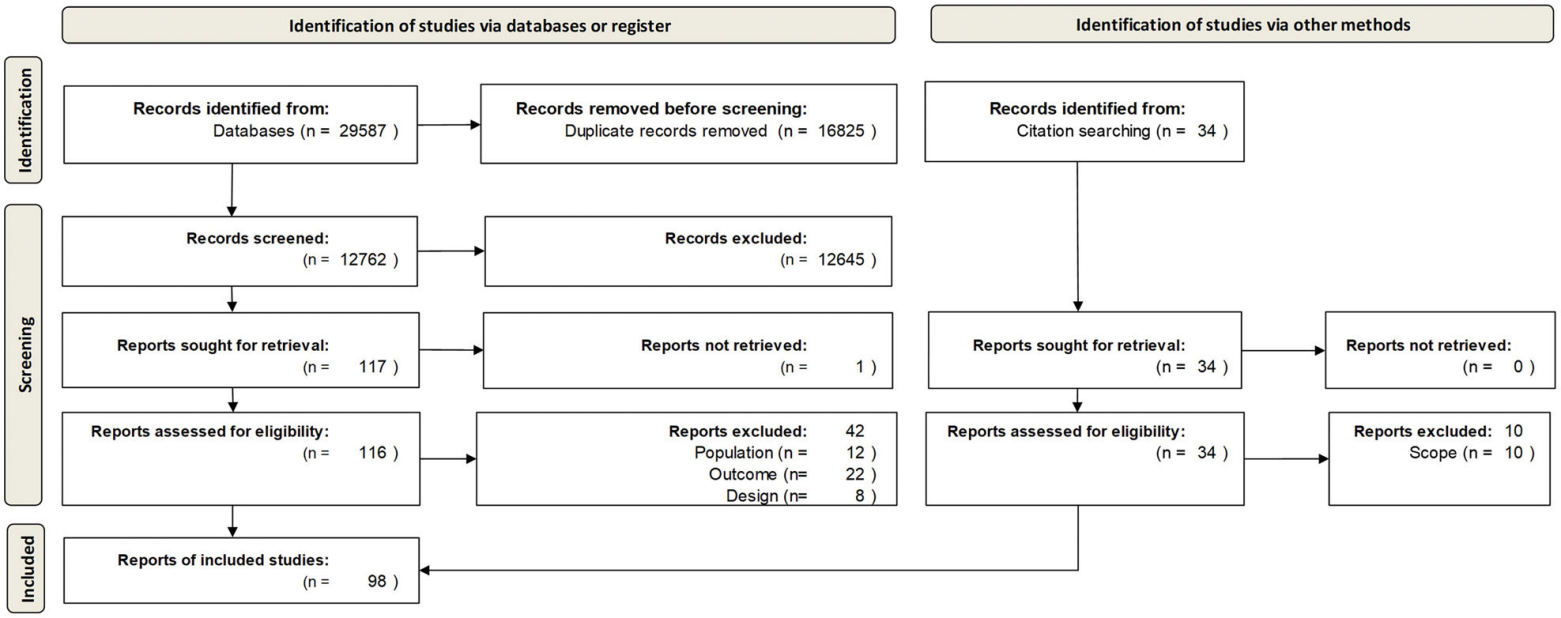
Flow diagram of literature search and selection process of the studies (PRISMA flow diagram).

The search through bibliographical databases yielded 29,587 hits, which was then reduced to 12,762 after removing duplicates. A total of 12,645 records were discarded following title and abstract screening. From the 117 full text articles that were retrieved and assessed for eligibility, 43 were excluded either because they did not provide any data or hypotheses concerning the aetiology of persistent symptoms or as they put forward hypotheses on long-term neurodegenerative diseases. In addition, 34 articles detected in the included studies’ reference lists or *via* a quick update search in PubMed were additionally retrieved and assessed, 24 of which were eventually included in our analysis. Of note, we also retained articles reporting post-mortem analyses, given that they provided insights into pathophysiological mechanisms. Nevertheless, their relevance for long COVID remains questionable, as they included patients who died from critical illness. As a result, 98 studies met our inclusion criteria and were included in the analysis. The selection of studies is summarized in Figure 1.

### Characteristics of included studies

We retrieved 54 articles that exclusively addressed hypotheses concerning potential underlying mechanisms of long COVID symptoms (see Table 1). Of these, 34 speculated on mechanisms that could specifically explain long COVID symptoms [10–43], whereas 18 focussed on organ injury developed during acute illness possibly resulting in persisting symptoms [44–61]. Concerning the latter, the suggested mechanism consisted of organ injury that complicated acute disease, possibly provoking persistent symptoms. The reported organ injuries were: stroke [46,52,55], myocardial infarction and fibrosis [41,43,49,51,55,57,59], acute encephalitis [43,50], neuromuscular disorders [40,41,43,46,48,56], renal failure [41,43,60,61], and hepatobiliary damages [43,44]. The other articles reported on endocrine disorders that were unrelated to organ damage, while including hypotheses on new-onset diabetes [62] and thyroid disorders [63].

**Table 1.** Pathophysiological mechanisms that may potentially be involved in long COVID symptoms.

System	Mechanisms
Neurological	Neuro-inflammation: persistent inflammatory process secondary to viral invasion or following the acute illness phase, resulting in brain microglia activation Activation of coagulation: microthrombosis impairing tissue vascularization and neurotransmission. Autoimmunity: aberrant immune response following the acute illness phase or molecular mimicry between SARS-CoV-2 and body antigens Metabolic brain disorders Residual virus particles: contributing to a low-grade smoldering inflammatory response Direct (stroke, Guillain Barré syndrome, myelitis) or indirect (hemodynamic and coagulation disorders, arrythmia) nervous system damage during the acute phase Activation of nerves (peripheral trigeminal nerve, nerve roots)
Fatigue	Neuro-inflammation Psychological factors Peripheral factors (musculoskeletal impairment) Environmental factors (social isolation temperature, humidity) Associated comorbidities Glymphatic-lymphatic system congestion Bioenergetic disorders in muscles due to mitochondria dysfunction
Smell and taste	Olfactory dysfunction due to viral invasion of olfactory mucosa (sustentacular cells)
Cardiovascular and coagulation	Endothelial dysfunction and subsequent coagulation activation – Endothelial invasion (ACE2 receptor) and consecutive coagulation activation and platelet/leucocyte attraction – Direct viral-induced activations of platelets (ACE2 receptor) – Neutrophil extracellular traps (NETs): inflammation-coagulation (Factor XII) – Direct complement activation (inflammation) – Pericytes invasion and endothelial cell injury (loss of endothelial homeostasis and integrity) – Antiphospholipid antibodies Cardiomyocyte impairments: viral invasion (through ACE2 receptor) Damages of the autonomic nervous system: intrathoracic chemo- and mechanoreceptors involved in cardiovascular reflexes
Respiratory system	Lung fibrosis Pulmonary vasculature damages (including microvessels) potentially leading to pulmonary hypertension. Damages in the autonomic nervous system (intrathoracic chemo and mechanoreceptors involved in the respiratory reflexes)
Immune system	Chronic dysregulated immune system activation: low-grade inflammation leading to multiple organ dysfunction. Mast cell activation syndrome Persistent smoldering infection Multi-system inflammatory syndrome in children (MIS-C)
Musculoskeletal system	Disruption of myocytes and fibroblast activation () Alteration of microcirculation in bones (hypercoagulability, leukocyte aggregation, and vessel inflammation) Autoimmunity and NETs activation in joints
Gastro-intestinal and hepato-biliary system	Post-infection gastro-intestinal dysfunction Microbiota alterations Hepato-biliary damage Autonomic nerve system disorder (gut motility disorders)
Renal system	Severity of critical illness Renal cell viral invasion Microangiopathy Renin-angiotensin-aldosterone pathway disorders Glomerulopathy
Endocrine system	Direct damage on the thyroid gland, subacute thyroiditis, low-T3 syndrome Viral invasion of pancreatic β cells
Multisystem Inflammatory Syndrome in Children (MIS-C)	Genetical predisposition host factors Uncontrolled T-cell immune response (triggered by SARS-CoV-2) Complement activation Molecular mimicry between antigens

Forty-four articles comprising patient data were retrieved. Ten of which were, in fact, post-mortem analyses that provided insights into pathophysiological mechanisms [64–73]. Studies were highly heterogeneous in nature, considering the time elapsed from infection to chronic symptoms. Furthermore, sample sizes were rather limited, while control groups were lacking in 11 studies [74–84]. In terms of inclusion criteria, the studies were heterogeneous, as well: 14 exclusively included patients that were hospitalized during the acute infection phase [74,85–97], 11 included both hospitalized and non-hospitalized patients [75,76,79,80,82,83,98–102], while another study exclusively comprised non-hospitalized patients [103]. Five studies did not mention the hospitalization status [77,78,81,84,104]. Examination techniques included magnetic resonance imaging (MRI), 18-fluoro-D-glucose positron emission tomography ([18F]FDG PET/CT), blood sample analysis and cytology/histology (mucosa brush cytological sampling, as well as skin and bowel biopsy).

### Central and peripheral nervous system

Persisting sequelae could either arise from neurological acute COVID complications resulting from brain damages [26], including stroke, encephalitis, and Guillain Barré syndrome, or from others factors related to hospitalization like delirium [43,58].

**Figure 2.**
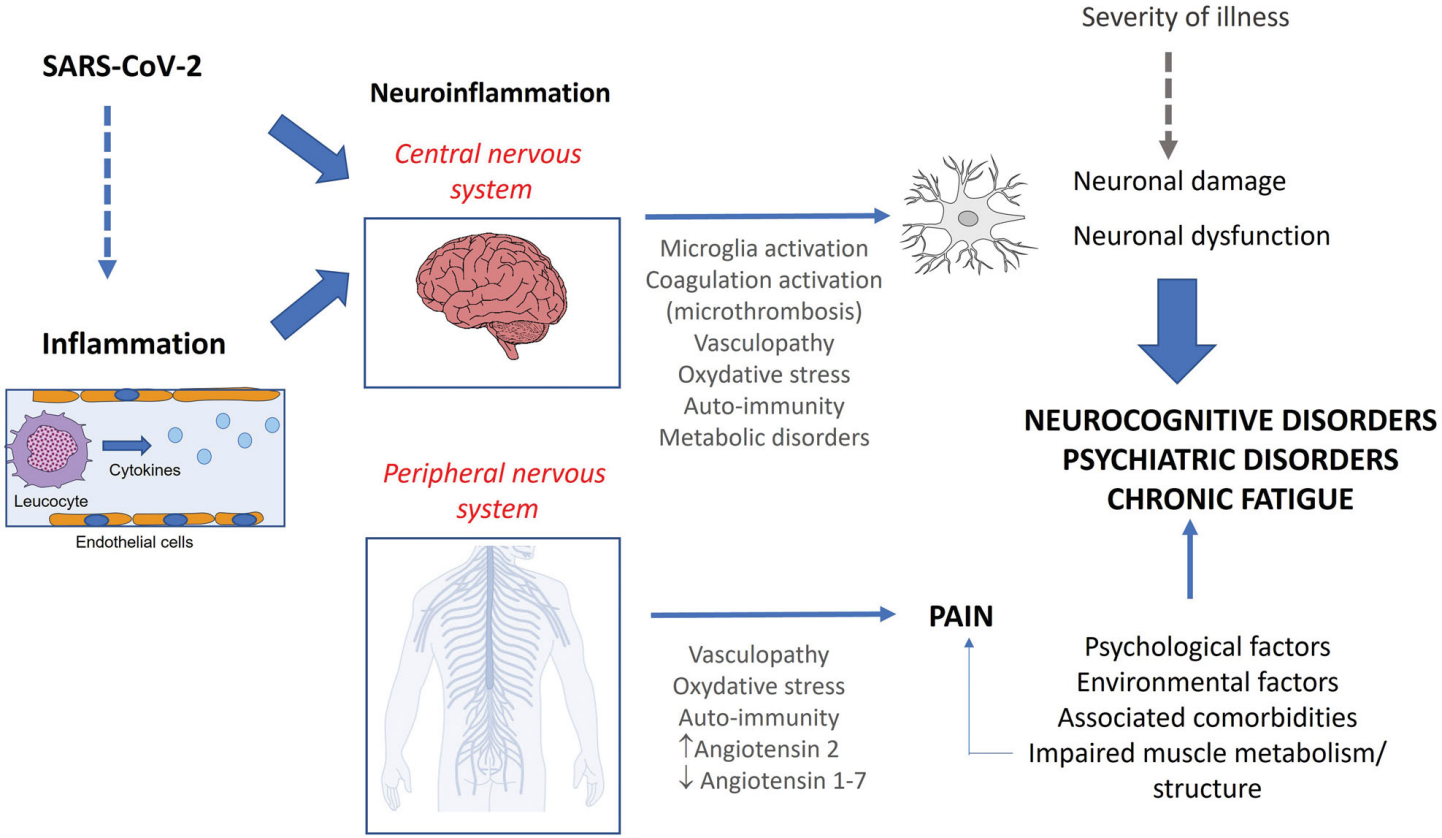
Different pathophysiological mechanisms potentially leading to persisting symptoms after COVID-19 (this figure has been included in the Belgian Health Care Knowledge Centre (KCE) report 344) [9].

Nevertheless, others neurological symptoms including cognitive or mental disorders, headache, and olfactory/gustatory dysfunction could depend on a pathophysiology that is independent from the acute phase. Indeed, the spreading of SARS-CoV-2 into the brain – through either the nasal cavity or bloodstream – has been suggested to trigger neuroinflammation. Many studies hypothesized on the role of sustained neuroinflammation in the onset of symptoms: through either an activation of microglia or auto-immune reactions, persistent neuroinflammation could account for neurocognitive impairment or mental health disorders [12,13,15,16,19–24,26–29,36–38,41,43,46,50,58]. In addition, local microthrombosis resulting from hypercoagulation or mitochondrial failure has also been suggested to contribute to symptoms (Figure 2). Two articles reported on structural alterations of the brain in symptomatic patients who recovered from COVID-19, based on MRI findings (diffusion tensing imaging and 3 dimensional T1-weighted sequences and pseudo-continuous arterial spin labeling) [90,100]. One of these reports highlighted structural modifications in several brain regions, including hippocampus, insular lobe, and olfactory cortex. Grey matter volume in various brain regions like hippocampus and cingulate gyrus was found correlated with memory or smell loss [90]. The other study reported a decrease in cortical thickness, combined with modifications in white matter microstructure, along with a decrease in regional cerebral blood flow in frontal and limbic regions [100]. In this line, another study identified that neuronal dysfunction markers were increased in patients who recovered from COVID-19, in comparison with healthy controls [99]. In addition, two autopsy studies [67,71] provided additional support in favour of the neuro-inflammation hypothesis, too.

In the same line, one study using electrophysiology techniques identified an impairment of GABA-ergic intracortical circuits [96]. However, studies that assessed the brain metabolism identified hypometabolic activity of the brain in patients presenting with various symptoms, including “brain fog” or anosmia [91,95,98]Trigeminal nerve or nerve root inflammation after COVID-19 have been proposed to be involved in headache and general pain [19,31,34,36,45,56].

Regarding the persistent smell and taste disorders, two studies reported on the role of neuroepithelial viral invasion and subsequent inflammation as possible mechanisms contributing to olfactory dysfunction [16,50]. Through histological assessment, a persistent inflammation within the olfactory neuroepithelium along with viral persistence was shown [77,78]. Likewise, biopsies demonstrated the persistence of SARS-CoV-2 in the cells of the taste buds of the tongue [104].

### Persistent fatigue

Persistent fatigue is a common symptom of long COVID with multifactorial causes. Abnormalities in the central nervous system like brain hypometabolism could be involved in its onset, which was evidenced among patients [98]. Muscle mitochondrial dysfunction could contribute to its physical dimension [30], while psychological and environmental factors were also likely to be involved [25]. One article suggested that a dysfunctional brain glymphatic drainage might lead to cerebrospinal fluid congestion, with toxin accumulation within the brain [31].

### Cardiovascular and coagulation disorders

Persisting cardiac abnormalities may be secondary to myocardial injury that occurred during acute infection, while being either consecutive to limited coronary perfusion or severe hypoxia [41]. However, the mechanisms perpetuating long-term cardiac symptoms may also arise from virus-induced microvascular disorders or myocardial inflammation. Four articles hypothesized on microvascular disorders [23,41,49,52,55], while six elaborated hypotheses in regard to induced heart abnormalities [23,41,49,51,52,55].

**Figure 3.**
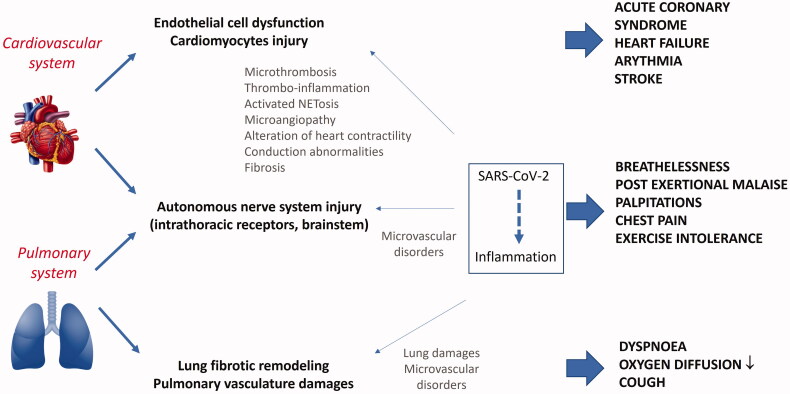
Mechanisms potentially involved in cardio-respiratory consequences after COVID-19 (this figure has been included in the Belgian Health Care Knowledge Centre (KCE) report 344) [9].

Systemic inflammation and invasion of cardiomyocytes by SARS-CoV-2 through angiotensin-converting enzyme 2 (ACE2) receptors have been proposed as potential mechanisms of heart dysfunction. Besides, owing to prolonged inflammation, the heart could subsequently undergo structural remodelling due to fibrosis pathway activation. Such evolution could be responsible for heart failure or arrythmia [43]. Besides, microvascular endothelial dysfunction within the heart and vessels could provoke microthrombi impairing appropriate vascularization (Figure 3). Four autopsy studies showed evidence of viral invasion within endothelial cells and cardiomyocytes with signs of inflammation and dysfunction [68,69,72,73], while manifestations of microcirculation abnormalities after recovery from COVID-19 were revealed by two studies [86,87].

Autoimmune disorders are another type of pathological consequences of COVID-19. The development of anti-phospholipid antibodies could possibly contribute to vascular inflammation and thrombotic complications. Such antibodies have been identified in COVID-19 patients upon the acute illness phase, which was associated with increased NETosis [49,52]. A study identified persistent inflammatory processes that were suggestive of vasculitis within large vessels, as based on [18F]FDG PET/CT performed in patients complaining about persisting symptoms after recovery [89].

Cardiovascular complaints could likewise result from nervous autonomic system disorders. Postural orthostatic tachycardia syndrome (POTS) could be caused by virus- or autoimmune-mediated dysfunction of the intrathoracic chemo- and mechano-receptors or of those located within the brainstem [10,14,19,36,43]. As such, antibodies against catecholamine receptors able to modulate heart frequency were identified, along with auto-antibodies against angiotensin 2 receptor and endothelin in patients with POTS (Figure 3) [81].

### Lung

Lung fibrosis is a potential long-term complication of COVID-19. Eight articles addressed the mechanisms leading to lung fibrosis, which generally occurs following severe respiratory inflammation and injury [32,41,43,47,53,54,57,59]. One of them emphasized that prolonged exposure to supplemental oxygen in most severe cases could lead to an increased oxidative stress within the lungs, thereby contributing in maintaining the inflammatory status while favouring the activation of fibrotic pathways [59]. Lung fibroproliferative damage was evidenced in autopsy specimens of patients who died from COVID-19 [65,66,70] in combination with endothelial damages and angiogenesis.

Long-term respiratory symptoms may be caused by lung vascular disorders. Indeed, one article emphasized the potential role of lung vascular damage in micro-vessels, possibly leading to pulmonary hypertension [47].

Four studies pointed out that dyspnoea, in the absence of pulmonary lesion, could hypothetically be related to inappropriate ventilation regulation resulting from autonomous nervous system disorders (potential damages in the intrathoracic reflex receptors or brainstem/cerebral cortical zones) [10,14,19,36].

Clinically, three studies focussed on the mechanisms underlying persisting lung inflammation, yet they exclusively included patients hospitalized during the early disease phase [75,80,85]. One study retrieved elevated inflammation and fibrosis biomarkers in a group of patients with chronic respiratory complaints, regardless of hospital admission [75]. Another evidenced signs of persisting lung inflammation using [18F]FDG PET/CT in patients who required mechanical ventilation [85]. One study suggested a relationship between respiratory impairments and dysregulated iron metabolism, possibly involved in organ damage [80]. In patients with lung sequelae in particular, a study showed metabolic abnormalities (involved with lung repair/fibrosis) correlating with lung diffusion capacity for carbon monoxyde [94].

### Immune system

Six articles suggested that underlying immune system disorders could lead to persistent symptoms of COVID-19 [11,18,19,32,35,39]. Two articles proposed a persistent smouldering infection as underlying mechanism [19,39]. Two articles considered that the mast cell activation syndrome could possibly contribute to long COVID symptomatology [11,18]. The third hypothesis put forth that sustained dysregulated immune system activation with subsequent chronic low grade inflammation could lead to pathological consequences like autoimmunity leading to organ dysfunction [19,32,35]. Four articles proposed mechanisms explaining the Multisystem Inflammatory Syndrome in children (MIS-C) [40–43].

Considering the patient level, thirteen articles supported the involvement of immune abnormalities [76,79,81,85,88,89,91,92,101–103,105,106], while one article focussed on MIS-C [97]. Long-lasting functional alterations of T-cells, with persistence of cytotoxic profile, were revealed by one study [88], while another one identified a decrease in dendritic cells revealed 7 months post-infection [106]. Residual excessive inflammation after infection was appraised by one study through the identification of proteins involved in inflammatory pathways (see Table 2) [103] Furthermore, auto-antibodies against immunomodulatory proteins or against tissues were clearly identified in three studies [81,101,105].

**Table 2.** Pathophysiological mechanisms identified in patients with long COVID symptoms.

System	Involved symptom(s)	Mechanisms
Neurology	Cognitive and mental health disorders Pain Headache Fatigue Anosmia/Agueusia Neuropathy	Functional brain disturbances Hypometabolic activity in various cerebral zones Reduced activity of the GABA inhibition Neuro-inflammation and brain microstructural modifications Micro-structural, volumetric and vascularization disorders
Smell and taste	Anosmia/Agueusia	Structural lesions in the olfactory and taste system at imaging and histology Injury in olfactory neuronal pathways Persistent inflammation of the neuroepithelium and with SARS-CoV-2 RNA identification Invasion and replication of SARS-CoV-2 in taste buds type II cells
Cardiovascular system	Fatigue Dyspnoea Chest pain	Persistent vascular inflammation Macrovascular vascular inflammation Microvascular inflammation: increased level of cytokines, circulating endothelial cells, coagulation activation microvascular retinal impairment (at autopsy, evidence of endothelial cells and cardiomyocytes viral invasion with signs of structural alterations) Auto-immunity: auto-antibodies able to modulate the cardiac frequency and vascular tone (acting as receptor agonists on the β2-adrenoceptor, the α1- adrenoceptor, angiotensin II AT1-receptor, angiotensin 1,7 and endothelin receptors) Persistent alteration of coagulation (sustained increased of D-dimer levels)
Respiratory	Dyspnoea Chest pain Cough	Persistent inflammation and dysregulated host response of lung repair Increased plasma biomarkers of lung inflammation and fibrosis (Lipocalin 2, Matrix metalloproteinase-7, Hepatocyte growth factor) Persisting inflammation in lungs, mediastinal lymph nodes, spleen, and liver Involvement of iron homeostasis disturbances in end-organ damage Relationship between metabolic abnormalities and lung sequelae
Gastro-intestinal system	No specific symptom	Gut microbiota modifications after recovery Decreases gut commensals with known immunomodulatory potential Perturbed composition of microbiota correlated inflammation biomarkers
Immune system	Multi-system symptoms	Persistent immune inflammatory response impairing organ functioning Remaining inflammation in blood samples analysis, long-lasting phenotypic and functional disorders of lymphocytes, decreased amounts of dendritic cells and persisting alterations of activation markers Signs of mild organ impairment at magnetic resonance imaging and [18F] FDG PET/CT Autoimmunity: auto-antibodies against the nociceptive receptors, immunomodulatory proteins (including cytokines, chemokines, complement components, and cell-surface proteins) and tissue components Persistence of the SARS-CoV-2 nucleic acids in tissues Multisystem Inflammatory syndrome in children (MIS-C)
Dermatological system	Skin disorders	At biopsy, presence of lymphocytic or neutrophilic infiltrates, endothelitis, microangiopathy, and microthrombosis

Of importance, the persistence of SARS-CoV-2 in the lower gastro-intestinal tract was revealed in asymptomatic subjects several months after COVID-19, suggesting that residual viral proteins within tissues could possibly be the basis for a persisting immune reaction [102].

Signs of diffuse and persistent immune inflammation impairing organ functioning was evidenced in four studies [76,85,91,92]. Three studies found signs of hypermetabolism in various organs using [18F]FDG PET/CT [85,89,91], while two others revealed signs of mild multi-organ impairment, as based on MRI [76,92]. Of these, one study demonstrated a correlation between the extent of extra-pulmonary abnormalities upon MRI and exercise intolerance and inflammation biomarkers, as well [92]. Lastly, owing to its immune system involvement, vitamin D has been investigated, yet without any relationship found between its concentration and lingering symptoms [79].

Specific features were identified for MIS-C in one article, including severe lymphopenia, low platelet counts, specific immune cell responses, and inflammation levels [97].

### Musculoskeletal and dermatological system

Four articles suggested that bone, joint, and muscle pain could be attributed to thrombo-inflammatory-mechanisms in relation with tissue injuries and autoimmune processes [40,41,43,48].

Numerous skin disorders have been reported. The underlying pathophysiology is still not well explored, and various abnormalities have been described on skin biopsies, including leucocyte infiltration, microthrombi, and vasculitis [82,83]. Their duration was investigated through an international register that included 234 patients. Based on its analysis, pernio lesions and livedo reticularis were reported to last longer than the other lesions, including morbilliform, urticarial, and papulo-squamous lesions [84].

### Other systems

According to one article, the cytokine storms of the acute phase of severe COVID-19 could induce hepato-biliary damage, possibly leading to chronic symptoms in the infection’s aftermath [44]. However, other authors suggested different mechanisms that were independent of the severity of acute COVID-19, three of which proposed either persisting low-grade gastro-intestinal tract inflammation [32,33,44] or autonomous nerve system dysfunction [32]. One study described persistent alterations of microbiota following recovery, which was characterized by a underrepresentation of commensals known for their immunomodulatory potential. A correlation between those disorders and inflammatory markers was evidenced [93].

Four articles reported on kidney damage [41,43,60,61]. Although acute kidney injury has been described in critically-ill patients who required renal replacement therapy at the acute COVID-19 phase [60], several different mechanisms have been incriminated in progressive chronic kidney disease. SARS-CoV-2 is able to invade several kidney cell types like podocytes or cells from the proximal tubule due to the expression of ACE2 on their surface [60]. In addition, endothelial dysfunction and microangiopathy have been proposed as underlying mechanisms, as have been alterations of the renin-angiotensin-aldosterone system [41,60]. One article described a possible glomerulopathy as the underlying pathophysiology of kidney damage after COVID-19, as observed for others viral infections [61].

Three articles focussed on endocrine disorders [43,62,63]. Although newly onset diabetes has been described in patients with COVID-19, a clear relationship between diabetes and SARS-CoV-2 has not yet been established. Since ACE2 is expressed on the pancreatic β cell, SARS-CoV-2 could damage these cells, thereby precipitating diabetes [62]. The thyroid can be affected by COVID-19 through different potential mechanisms. Direct damage to the thyroid could occur through direct viral invasion, given that ACE2 is expressed on the gland’s cells, or indirect damage through the release of cytokines at the early illness phase, which could induce inflammation within the thyroid gland [63]. On the other hand, other thyroid conditions have been described like the low triiodothyronine (T3) syndrome in severely ill hospitalized patients or late-onset autoimmune subacute thyroïdis [63].

## Discussion

Presently, the evidence about what causes long COVID is still limited, and the underlying mechanisms thus remain ambiguous. Many hypotheses have been put forth by articles that reviewed the potential pathophysiology of long COVID [8,32,41,107,108]. In this review based on a systematic approach, we synthesized the currently available information, with particular emphasis on the research conducted on long COVID patients. Since the clinical spectrum is highly heterogeneous, we have made a distinction between symptoms related to organ dysfunction upon acute COVID-19 and the pathophysiology unrelated to organ damage possibly involved in lingering symptoms. Although organ injuries developed during the acute phase can account for the long COVID symptomatology, there is now compelling evidence that patients who experienced mild or moderate forms of acute COVID-19 can still present symptoms that are unassociated with organ dysfunctions incurred during the early phase [2,6,109].

Our review has provided arguments to support several mechanisms potentially involved in long COVID. First, virus-driven cellular alterations related to its neurotropism have been suggested to possibly contribute to the pathophysiology of long COVID. This mechanism could account for olfactive disorders or manifestations related to autonomous nervous system dysfunction. Second, a dysregulated immune reaction in response to either initial infection or occult viral persistence is thought to possibly provoke deleterious disorders, including autoimmune manifestations, coagulation and fibrosis pathway activation, or metabolic disturbances. Importantly, empirical studies on patients gave rise to the possibility of organ dysfunctions through inflammatory or autoimmune mechanisms. Moreover, several studies brought about elements regarding the hypothesis of persistent and occult virus presence, as based on the identification of viral particles in several organs after the acute infection [77,78,102,104]. Hence, our review clearly revealed that the clinical picture of long COVID could presumably not be ascribed to a single pathophysiological mechanism, and that responsible mechanisms were probably numerous and intertwined.

After the end of our research, new aetiological elements were published, providing more insight into those inflammatory and auto-immune aspects of long COVID. Antibodies against the ACE2 receptor have been identified in COVID-19 convalescent patients. The authors demonstrated that the activity of ACE2 was reduced. This could lead to an increase in angiotensin II, thereby inducing a proinflammatory state [110].

Articles reporting patient data are still highly heterogeneous, exhibiting several limitations. First, the characteristics of patients included in studies vary considerably. In addition, the patients included presented a wide variety of symptoms that potentially occur in different combinations. Since long COVID generally manifests as a combination of disorders, some authors focussed on a better description of subtypes based on long COVID complaints [3]. Interestingly, the narrative review conducted by Yong et al. proposed six distinct phenotypes, including multi-organ sequelae (from acute illness), chronic fatigue, pulmonary fibrosis sequelae, orthostatic syndromes, post-intensive care syndrome, and medical/clinical sequelae (related to chronic health conditions) [3]. However, research on the underlying mechanisms is commonly conducted regardless of symptom types or in patient samples with mixed symptoms. Furthermore, the initial severity and care level of targeted patients was shown to vary considerably. Conversely, several studies also included asymptomatic patients [81,99,102,103,106]. It should be noted that study designs were heterogeneous. Most studies included small sample sizes, and the time-points of inclusion differed across studies. Due to the exploratory design of pathophysiological studies, the selection process concerning patients was not necessarily reported; which represents a substantial bias. Moreover, many studies included healthy volunteers as control group rather than COVID-19 patients who did not develop long COVID.

It must also be emphasized that some results prove to be difficult to interpret. For instance, the studies suggesting organ impairment indices based on MRI findings associate these abnormalities with either symptoms or biomarkers, without explaining the underlying mechanisms [76,92]. Furthermore, the relationship between detected abnormalities and symptoms or clinical conditions was not always examined.

Likewise, symptoms such as fatigue are often difficult to assess, given that its causes are complex and multifactorial in nature. In this setting, long COVID could share some similar elements of post-infectious fatigue, which is observed after viruses like Influenza or other coronaviruses [111] or with Myalgic Encephalomyelitis/Chronic Fatigue Syndrome (ME/CFS). In a large cross survey, Davis et al. reported that typical ME/CFS symptoms like post-exertional malaise were experienced following COVID-19. On the other hand, a recent systematic review including 21 studies noticed that major criteria symptoms of ME/CFS, including fatigue, post-exertional malaise, and reduced daily activity, were reported by the majority of patients included in long COVID studies [112]. Despite the possible overlap between both conditions [107,112], notable differences exist. A recent study compared ME/CFS symptoms and their duration through a survey using the Depaul Symptom Questionnaire with a list of COVID-19 symptoms [113]. Long COVID patients were initially more symptomatic than MCE-CF patients with respect to the immune (respiratory symptoms, fever and lymph nodes) and orthostatic domains, whereas ME/CFS patients displayed more gastrointestinal and neurocognitive symptoms. Long COVID patients reported an improvement in most symptoms over time except for neurocognitive symptoms, while the illness duration was longer in MCE-CF patients. Unlike for MCE-CF, the infection as a trigger was generally documented for long COVID. Likewise, the research into long COVID is still inceptive and in spite of clinical similarities, it is too soon to draw sound conclusions about the underlying pathophysiological mechanisms.

When investigating the processes underlying long COVID, a possible pitfall is that long COVID patients who were asymptomatic at the acute phase could be overlooked and thus not be included in studies. Owing to this lack of awareness and monitoring, distinct pathophysiological pathways may be overlooked. Indeed, a weaker immune response could certainly play a role in long COVID [114], while a longer duration of viral shedding could hypothetically contribute to long-term immunity disorders [1,6].

Our review has several limitations. Firstly, the literature on long COVID is rapidly evolving, and keeping up to date is subsequently difficult. However, our search included many databases and, although the actual search was only performed until May 2021, a manual screening regularly performed until August 2021 enabled us to update the evidence. Moreover, some articles published after the end of our search were included in the discussion section. Secondly, the retrieved evidence could be flawed because the quality of evidence was not assessed. Articles based exclusively on hypotheses could not be assessed under standard conditions, whereas many studies on patients presented shortcomings. Owing to the urgent need to understand how to manage long COVID, most studies that were aimed at unravelling the disease pathophysiology omitted key components of methodological quality. Ongoing trials assessing therapeutic interventions are likely to further shed light on the mechanisms involved in long COVID.

## Conclusion

Up till now, research on the pathophysiological mechanisms underlying long COVID has mainly focussed on the acute COVID-19 phase and its subsequent organ dysfunctions. A lot of the research has been focussed on hypothesis generation. Empirical studies are highly heterogeneous in nature, thus rendering their assessment complicated. Grasping the pathophysiology of long COVID remains puzzling, since the clinical spectrum is highly variable and affects many organs [1,8,115]. In addition, since symptoms abate over time, the pathophysiological exploration must be performed at different time-points in order to better identify the timeline of mechanisms and natural history of long COVID. Moreover, the role of SARS-CoV-2 in the onset of neurodegenerative diseases or cancers is still unsure and requires more research. Only a robust understanding of the pathophysiology of long COVID will enable us to appraise and manage these disease consequences [116,117].

## Author contributions

DCZ and KVH conceived the design of the study and performed the analysis. KVH supervised the study. PC designed and performed the literature search strategy, and the organization of the database. DCZ wrote the manuscript with the assistance of KVH, PC and IC. All authors participated to the research process and manuscript redaction, while provided critical feedback. All authors approved the submitted version.

## Data Availability

Data sharing is not applicable to this article, given that no new data were created or analysed in this study.
